# Prebiotic, Probiotic, and Synbiotic Consumption Alter Behavioral Variables and Intestinal Permeability and Microbiota in BTBR Mice

**DOI:** 10.3390/microorganisms9091833

**Published:** 2021-08-29

**Authors:** Jodi E. Nettleton, Teja Klancic, Alana Schick, Ashley C. Choo, Ning Cheng, Jane Shearer, Stephanie L. Borgland, Jong M. Rho, Raylene A. Reimer

**Affiliations:** 1 Faculty of Kinesiology, University of Calgary, Calgary, AB T2N 1N4, Canada; jenettle@ucalgary.ca (J.E.N.); klancic.teja@gmail.com (T.K.); acchoo@ucalgary.ca (A.C.C.); jshearer@ucalgary.ca (J.S.); 2 International Microbiome Centre, Cumming School of Medicine, University of Calgary, Calgary, AB T2N 4N1, Canada; Alana.Schick@bcchr.ca; 3 Alberta Children’s Hospital Research Institute (ACHRI), Cumming School of Medicine, University of Calgary, Calgary, AB T2N 4N1, Canada; ncheng@ucalgary.ca (N.C.); jrho@health.ucsd.edu (J.M.R.); 4 Department of Biochemistry & Molecular Biology, Cumming School of Medicine, University of Calgary, Calgary, AB T2N 4N1, Canada; 5 Hotchkiss Brain Institute, University of Calgary, Calgary, AB T2N 4N1, Canada; slborgla@ucalgary.ca; 6 Departments of Neurosciences & Pediatrics, University of California San Diego, Rady Children’s Hospital San Diego, San Diego, CA 92123, USA

**Keywords:** autism, microbiota, BTBR mouse, prebiotic, probiotic, synbiotic, sociability, ultrasonic vocalization, repetitive behavior, permeability

## Abstract

Given that prebiotics have been shown to improve gut microbiota composition, gastrointestinal symptoms and select behaviors in autism spectrum disorder (ASD), we hypothesized that prebiotic supplementation would improve sociability, communication, and repetitive behaviors in a murine model of ASD. We also examined the effect of a synbiotic (probiotic + prebiotic). Juvenile male BTBR mice were randomized to: (1) control; (2) probiotic (1 × 10^10^ CFU/d *Lactobacillus reuteri* RC-14^®^; now known as *Limosilactobacillus reuteri*); (3) prebiotic (10% oligofructose-enriched inulin); (4) prebiotic + probiotic (n = 12/group) administered through food for 3 weeks. Sociability, communication, repetitive behavior, intestinal permeability and gut microbiota were assessed. Probiotic and symbiotic treatments improved sociability (92 s and 70 s longer in stranger than empty chamber) and repetitive behaviors (50% lower frequency), whereas prebiotic intake worsened sociability (82 s less in stranger chamber) and increased the total time spent self-grooming (96 s vs. 80 s CTR), but improved communication variables (4.6 ms longer call duration and 4 s higher total syllable activity). Mice consuming probiotics or synbiotics had lower intestinal permeability (30% and 15% lower than CTR). Prebiotic, probiotic, and symbiotic treatments shifted gut microbiota to taxa associated with improved gut health. *L.reuteri* may help alleviate ASD behavioral symptom severity and improve gut health. The potential use of prebiotics in an ASD population warrants further research.

## 1. Introduction

Autism spectrum disorder (ASD) is a heterogeneous neurodevelopmental disorder that is diagnosed in early life based on three core symptoms: communication deficits, impaired sociability, and repetitive or restricted behavior [1]. Currently, it is estimated that ASD impacts nearly 1 in 59 children in the United States, and is four-times more common in males than females [2]. The exact cause(s) of ASD is/are unknown; however, potential contributors that have been studied are genetics, environmental factors including intrauterine insults, and health conditions, such as epilepsy and muscular dystrophy [3].

A commonly reported comorbidity of ASD is gastrointestinal (GI) distress, and this has been estimated to impact 10% [4] to 70% [5] of the ASD population. Moreover, GI distress has been correlated with autism severity [6,7], but may be underreported due to the limited communication and verbal capacities of individuals with ASD, thus manifesting in increased problem behavior, such as aggression and self-injury [8].

The “leaky gut” hypothesis has been examined as a potential mechanism behind the higher prevalence of GI distress in ASD populations. Intestinal epithelial cells are joined together by multi-protein complexes called tight junctions [9], and prevent intestinal lumen contents from passing into surrounding spaces and circulation. However, when tight junction proteins are disturbed and weakened, intestinal hyperpermeability results, and commensal microorganisms may pass through the epithelium and disrupt other layers of the gut, invoking innate immune responses such as low-grade inflammation [10]. Abnormal intestinal permeability has been observed in human ASD [11].

Although there is no clear gut microbiota signature in ASD, studies have shown a distinctly different profile in ASD versus controls, and some studies have shown an alteration in *Clostridiale* species [6,12,13,14,15,16,17]. Observed changes in microbiota may account for the severity of symptoms experienced in ASD. For example, one study found significantly higher abundances of Bacteroidetes and *Bacteroides vulgatus* in ASD, which have been implicated in the development of irritable bowel disorders [18,19]. Interestingly, in an animal model of the core behavioral deficits in autism, the BTBR mouse, an increase in Bacteroidetes abundance has been observed, and is positively correlated with measures of intestinal permeability [20]. Kang and colleagues found that oral and rectal fecal microbiota transplants (termed “microbial transfer therapy”, or MTT) in children with ASD experiencing GI distress altered behavior and significantly improved GI symptoms, alongside changes in microbiota composition [21]. Improvements in behavior and GI function were sustained two years following treatment, and gut microbiota diversity remained significantly higher compared to baseline [22]. Thus, if comorbidities of ASD can be influenced by the gut microbiota, this could be an attractive non-invasive therapeutic target for treatment.

One ASD treatment attracting attention recently is the use of probiotics, live microorganisms that confer health benefits to the host if consumed in adequate amounts [23]. One report estimated that 36.5% of Canadian doctors encouraged probiotic supplementation for patients with ASD [24], and probiotic treatment in ASD has shown improvements in behavioral outcomes, as well as improvements in gut microbiota in humans and mice [12,25,26].

Prebiotics are compounds that resist host digestion and are selectively used by host microorganisms to confer health benefits upon the host [27], including reducing intestinal permeability [28]. Studies have found that prebiotics have the potential to reduce GI distress in individuals with ASD, but less is known about the effects on ASD behavior. A synbiotic is a product containing both prebiotics and probiotics [29], and it has been shown in rats to improve intestinal permeability and gut microbiota profiles [30], and in children diagnosed with ASD (n = 8) to reduce lethargy and some GI symptoms [31].

Findings that prebiotics and synbiotics improve intestinal permeability and gut microbiota profiles may have important implications for the health of ASD populations. Thus, the aim of the current study is to examine the effects of prebiotic, probiotic, and synbiotic consumption on sociability, repetitive behavior, and communication, alongside intestinal permeability and gut microbiota profiles, in a murine model of ASD.

## 2. Materials and Methods

### 2.1. BTBR Rodent Care and Intervention

The University of Calgary Animal Care Committee granted ethical approval for the current study (Protocol #: AC13-0313) and all experiments were performed in accordance with the Guide to the Care and Use of Laboratory Animals by the Canadian Council on Animal Care. At postnatal day (PND) 23, forty-eight male BTBR mice were randomized to one of four diets for 3 weeks: (1) control (CTR; AIN93 diet, Dyets^®^, Bethlehem, PA, USA); (2) probiotic (PRO; 1 × 10^10^ colony forming units (CFU)/d *Lactobacillus reuteri*, RC-14^®^;–Chr. Hansen, Hørsholm, Denmark); (3) prebiotic (PRE; 10% oligofructose-enriched inulin, Orafti^®^Synergy1, Beneo, Mannheim, Germany); (4) PRE + PRO (SYN). *L. reuteri* (now known as *Limosilactobacillus reuteri*) was selected as the probiotic based on work showing it improved sociability in BTBR mice [26] and oligofructose-enriched inulin was selected based on work showing it improves intestinal permeability [28]. Mice were housed in groups of three per cage in the Health Sciences Animal Resource Centre, University of Calgary, in a temperature- (21–22 °C) and humidity (41–44%)-controlled room with a 12 h light/dark cycle (light cycle: 0700-1900h). Food was prepared weekly in powder form, stored in the refrigerator, and changed in the mouse cages every two days. Body weight was measured weekly. Food intake was measured for five consecutive days each week over the duration of the study. Baseline measurements were recorded 24 h prior to treatment initiation (PND 22). A schematic of the study is provided in Supplementary Figure S1.

### 2.2. Behavior Tests

#### 2.2.1. Repetitive Behavior

All behavior tests were conducted on postnatal days (PNDs) 42 and 43 and mice were given a 60 min habituation period in the new room before testing. The tests were performed following the 3-week dietary intervention to reduce stress, which may alter behavioral outcomes. Testing was performed in the dark under infrared illumination to reduce anxiety between 0800 and 1400 h Mountain Standard Time (MST). A white noise machine was used during behavioral testing to minimize the stress and impact of sudden noises from the environment. To examine repetitive behavior, mice were placed in a sterilized chamber (dimensions of 23 (length) × 40 (width) × 30 (height) cm) for a 10 min habituation phase followed by a 10 min testing phase. Self-directed repetitive behavior was recorded using a camera, and total duration, frequency, and average grooming time were assessed by a trained researcher.

#### 2.2.2. Sociability

Immediately following repetitive behavior testing, sociability in BTBR mice was tested using a three-chambered Plexiglass apparatus for three consecutive 10 min phases. Tests were conducted between 0800 and 1400 h MST in the dark under infrared illumination. During the first phase, mice were habituated to the center chamber for 10 min, followed by a 10 min habituation phase in all three empty chambers. Finally, two wire cages were introduced to the side chambers—one wire cage was empty, while the other contained a “stranger” BTBR mouse. Time spent in each chamber was recorded using Ethovision^®^ XT tracking software. The plexiglass chamber was sterilized with 70% ethanol and wiped dry using paper towel between each animal test.

#### 2.2.3. Communication

Communication testing occurred between 1800 and 2000 h MST on PND 43. BTBR male mice were separated and singly housed for 3 h, then a female BTBR mouse was introduced to the male mouse’s home cage. Ultrasonic vocalization (USV) calls were recorded for 5 min with a microphone extended 20 cm above the cage using Aviosoft software. Vocalization frequencies lower than 25 kHz were filtered through a high-pass filter and manually assessed for background noise using the Mouse Ultrasonic Profile ExTraction software [32] on MATLAB^®^. USV calls were manually classified into the following call categories: complex, unstructured, two-syllable, upward, downward, short, flat, chevron, composite, and frequency step calls based on previous research [33].

### 2.3. Intestinal Permeability

Intestinal permeability was evaluated in vivo using fluorescein isothiocyanate-dextran-4000 daltons (FITC-4000) (Sigma Aldrich, Oakville, Canada) on PND 45. Following 5 h food deprivation (0700–1200 h MST), mice received 500 mg/kg FITC-4000 via oral gavage. Blood (120–150 µL) from the saphenous vein was collected into a tube containing 1.25 μL ethylenediaminetetraacetic acid (EDTA) 1 h post-gavage and centrifuged at 4 °C for 3 min at 12,000× *g*. Plasma samples were diluted in equal volumes of PBS and 50 µL were loaded onto a 96-well plate alongside serially diluted standards. The plate was read on a Microplate Fluorescence reader (FLX 800) at an excitation wavelength of 485 nm and emission wavelength of 535 nm.

### 2.4. Gut Microbiota Sequencing

Fresh fecal matter was collected at PND 42 and stored at −80 °C until analysis. Genomic DNA was extracted from fecal samples using bead-beating disruption and a FastDNA Spin Kit for Feces (MP Biomedicals, Lachine, QC, Canada) and quantified using a PicoGreen DNA quantification kit (Invitrogen, Carlsbad, CA, USA). Then, 16 S rRNA gene sequencing was performed on the MiSeq Illumina platform at the Centre for Health Genomics and Informatics (University of Calgary) with amplification of the V3–V4 regions according to our previously published methods [34]. Raw fastq files were processed using a custom pipeline. Cutadapt (version 1.16) [35] was used to remove standard Illumina V3–V4 primers from the reads as well as reads shorter than 50 bp. Quality filtering was performed using the filterAndTrim function from the R package dada2 (version 1.8.0), with the following criteria: (1) forward and reverse reads were truncated at a length of 265 bp and 245 bp, respectively; (2) the first 8 bp of each read was removed; (3) after truncation, forward and reverse reads with higher expected errors (EE = sum(10^(−Q/10)) than 2 and 5, respectively, were discarded. A table of amplicon sequence variants (ASVs) was generated using the standard dada2 workflow: generating an error model of the data, inferring sequence variants, merging forward and reverse reads, generating a count table, and removing chimeric sequences. Taxonomic classifications were assigned to ASVs using the dada2::assignTaxonomy function, using the Silva 132 database as a reference.

### 2.5. Statistical Analysis

Sample size was determined based on previous research outlining the ideal number of animals required for behavioral phenotyping in autism-based research [36]. All data presented are mean ± SEM unless otherwise specified. For outcomes with repeated measures over time, a three-way mixed ANOVA was employed to assess for the main effects and interaction effects of the *between-subject* factors (prebiotic and probiotic) with time as the *within-subject* factor. For outcomes with single time points, a two-way ANOVA was used to assess the main effects of prebiotic or probiotic treatment and their interaction. If an interaction was observed, a one-way ANOVA and Bonferroni *post-hoc* was used to determine where amongst the four groups the difference(s) existed and to correct for multiple comparisons. Statistical calculations were performed using IBM^®^ SPSS^®^ Statistics Version 24. Differences were considered significant if *p* < 0.05. For the correlational analysis, results were considered significant if *p* < 0.01 to correct for multiple comparisons using the Bonferonni method.

## 3. Results

### 3.1. Body Weight and Food Intake

**Figure 1:**
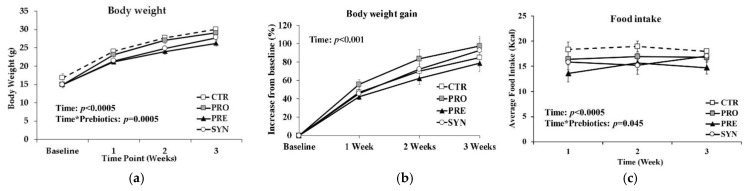
Body weight gain and food intake of BTBR mice consuming control, probiotic, prebiotic, or synbiotic diets for three weeks. (**a**) Body weight over time; (**b**) body weight gain (% increase from baseline); (**c**) average daily food intake (kcal/day). Values or mean ± SEM, n = 12/group. *p*-values presented in the figure represent the main effects and interaction effects from the repeated measures ANOVA. CTR, control; PRO, probiotic; PRE, prebiotic; SYN, synbiotic.

The body weight of mice increased over time (Figure 1a; *p* = 0.0005) from a mean baseline of 15.4 ± 0.7 g, but there were no significant differences in the percentage body weight gain between the groups consuming control (85 ± 10% from baseline at 3 weeks), probiotic (98 ± 10%), prebiotic (79 ± 9%) or synbiotic (93 ± 13%) diets (Figure 1b). There was a significant time×prebiotic effect (*p* = 0.045) of food consumption, with prebiotic mice consuming less (14.7 ± 1.2 g per day) compared to CTR (18.0 ± 0.2 g), PRO (16.8 ± 0.6 g) and SYN (17.0 ± 1.3 g) (Figure 1c).

### 3.2. Social Behavior

**Figure 2:**
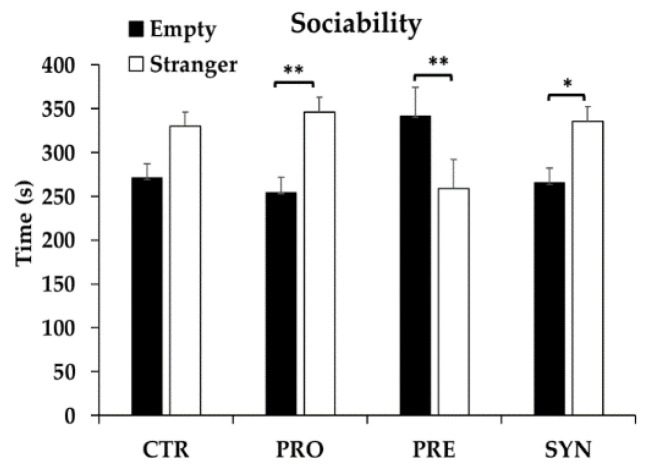
Sociability behavior of BTBR mice at postnatal day 43–44 following consumption of a control, probiotic, prebiotic, or synbiotic diet for three weeks. Values or mean ± SEM, n = 9–10/group. * *p* ≤ 0.05; ** *p* < 0.025; CTR, control; PRO, probiotic; PRE, prebiotic; SYN, synbiotic.

When comparing time spent in the chamber containing a stranger mouse with time spent in empty chambers, PRO and SYN mice spent significantly more time with the stranger mouse (91.8 s and 70.2 s more, respectively) (*p* < 0.05), whereas PRE mice spent significantly less time (82 s less) (*p* = 0.011); no difference was observed for control mice (59 s) (*p* = 0.063) (Figure 2).

### 3.3. Communication Variables

**Figure 3:**
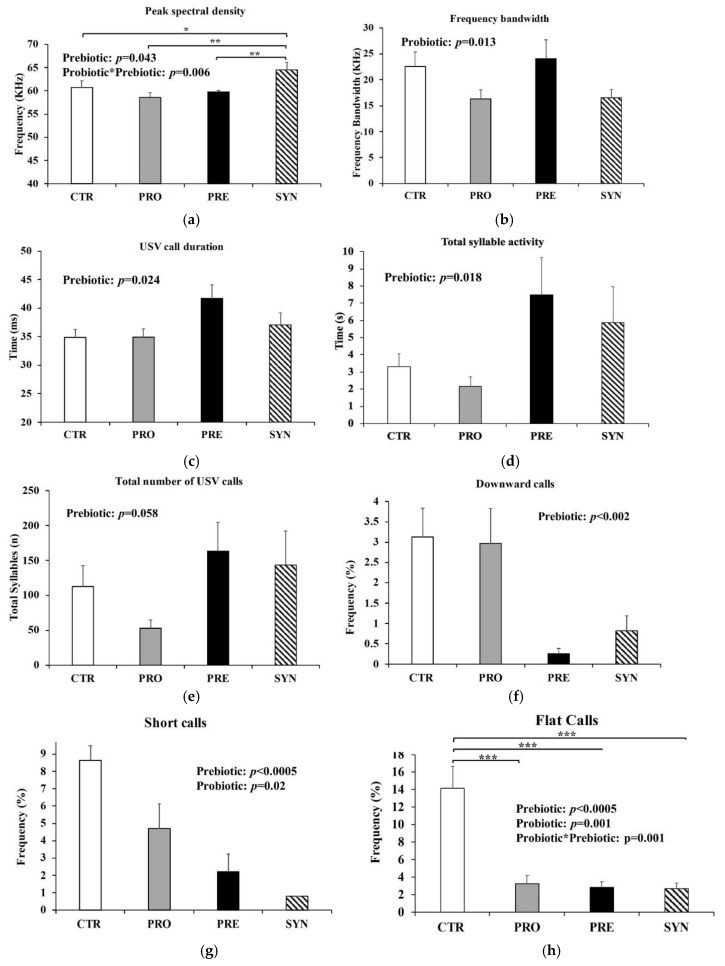
USV calling behavior of BTBR mice during a five-minute testing period at postnatal day 43 after consuming a control, probiotic, prebiotic, or synbiotic diet for three weeks. (**a**) Peak spectral density; (**b**) frequency bandwidth; (**c**) total USV call duration; (**d**) total syllable activity; (**e**) total number of USV calls; (**f**) frequency of downward-style calls; (**g**) frequency of short-style calls; (**h**) frequency of flat-style calls. Values or mean ± SEM, n = 7–8/group. P-values presented in the figure represent the main effects and interaction effects from the two-way ANOVA. * *p* ≤ 0.05; ** *p* < 0.025; *** *p* < 0.0005. CTR, control; PRO, probiotic; PRE, prebiotic, SYN, synbiotic.

Power spectral density (PSD) was calculated to determine the frequency at peak call (kilohertz, kHz) emitted by the mice [32,37], also referred to as peak frequency. There was a significant interaction between prebiotic and probiotic for PSD (*p* = 0.006), whereby SYN mice had significantly greater PSD (64.5 ± 1.6 KHz) compared to all other groups (CTR 60.7 ± 1.4; PRO 58.6 ± 1.0; 59.8 ± 0.3 KHz) (*p* < 0.05) (Figure 3a), suggesting that SYN mice emit USV of greater frequencies (kHz). Mice consuming probiotics had 30% lower mean frequency bandwidth of USV calls compared to mice not consuming probiotics (*p* = 0.013) (Figure 3b). Frequency bandwidth is the distance between the minimum and maximum frequency in each vocalization recorded [38]. Prebiotic consumption was associated with a greater mean call duration of 4.63 ± 1.9 milliseconds (msec; *p* = 0.024) (Figure 3c) and a total syllable activity that was higher by nearly four seconds (*p* = 0.018; Figure 3d). Mice consuming prebiotics tended to have a greater total number of USV calls (mean 153 calls) over the 5 min testing period, but this was not significant (*p* = 0.058) (Figure 3e). Prebiotics reduced the number of downward calls (PRE 0.3 ± 0.1%; SYN 0.8 ± 0.4%; CTR 3.1 ± 0.7%; PRO 3.0 ± 0.9%) (*p* < 0.002) (Figure 3f), and prebiotics and probiotics both independently reduced the percentage of short calls by over 50% (*p* = 0.02) (Figure 3g). There was a significant interaction between prebiotics and probiotics for flat calls (*p* = 0.001), whereby PRE (2.8 ± 0.6%), PRO (3.3 ± 0.9%) and SYN (2.7 ± 0.6%) all resulted in a reduced percentage of flat USV calls compared to CTR (14.2 ± 2.5%) (*p* < 0.0005) (Figure 3h).

### 3.4. Repetitive Behaviors

**Figure 4:**
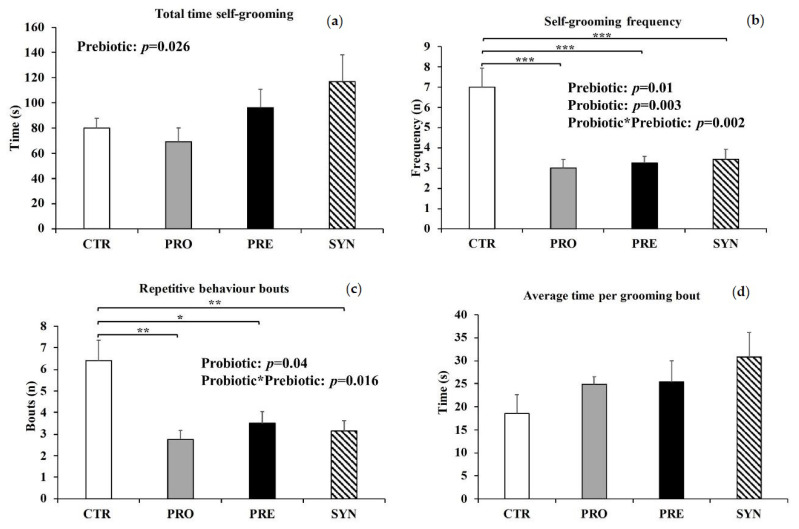
Self-grooming behaviors at postnatal day 42–43 in BTBR mice consuming either a control, probiotic, prebiotic, or synbiotic diet for three weeks. (**a**) Total time spent self-grooming; (**b**) self-grooming frequency; (**c**) repetitive behavior bouts; (**d**) average time per grooming bout. Values or mean ± SEM, n = 8–10/group. P-values presented in the figure represent the main effects and interaction effects from the two-way ANOVA. * *p* ≤ 0.05; ** *p* < 0.025; *** *p* < 0.0005. CTR, control; PRO, probiotic; PRE, prebiotic, SYN, synbiotic.

There was a main effect of prebiotics on total time spent self-grooming (*p* = 0.026), whereby mice consuming prebiotics (PRE and SYN) spent on average 32 s longer performing repetitive grooming behaviors during the entire ten-minute testing period (Figure 4a). There was a significant interaction between prebiotics and probiotics in terms of frequency of self-grooming (*p* = 0.002) (Figure 4b), whereby mice consuming prebiotics and/or probiotics had a nearly four-fold lower grooming frequency compared to mice not consuming prebiotics or probiotics. We further divided grooming frequencies into bouts and interrupted bouts. Bouts of grooming are described as grooming sessions that are interrupted by at least six seconds of other activities or inactivity, and interrupted bouts are grooming sessions that are separated by less than six seconds of inactivity or activities other than grooming [39]. There was a significant interaction between probiotic and prebiotic consumption (*p* = 0.016), whereby BTBR mice consuming prebiotics and/or probiotics (PRE 3.5 ± 0.5; PRO 2.8 ± 0.4; SYN 3.1 ± 0.5 bouts) had significantly lower bouts of repetitive grooming than CTR (6.4 ± 0.9 bouts) (*p* < 0.05) (Figure 4c), and tended to spend approximately 40% more time per grooming bout (Figure 4d); however, this outcome was not significant. No differences in interrupted bouts were observed between all groups (data not shown).

### 3.5. Intestinal Permeability

**Figure 5:**
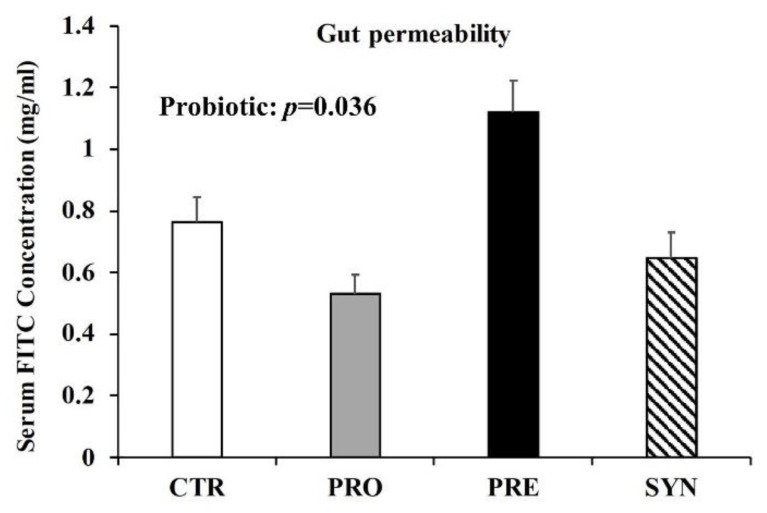
The impact of three-week prebiotic, probiotic, or synbiotic consumption on intestinal permeability, measured as serum FITC-4000 concentration. Values or mean ± SEM, n = 9/group. CTR, control; PRO, probiotic; PRE, prebiotic, SYN, synbiotic.

There was no statistically significant interaction between prebiotic and probiotic consumption in terms of FITC-4000 serum concentration. There was, however, a significant main effect of probiotics, whereby mice consuming probiotics (PRO 0.53 ± 0.06 and SYN 0.65 ± 0.08 mg/mL FITC) had reduced serum FITC-4000 (*p* = 0.036) (Figure 5), suggesting that probiotic consumption may improve the integrity of the gut barrier.

### 3.6. Fecal Microbiota

**Figure 6:**
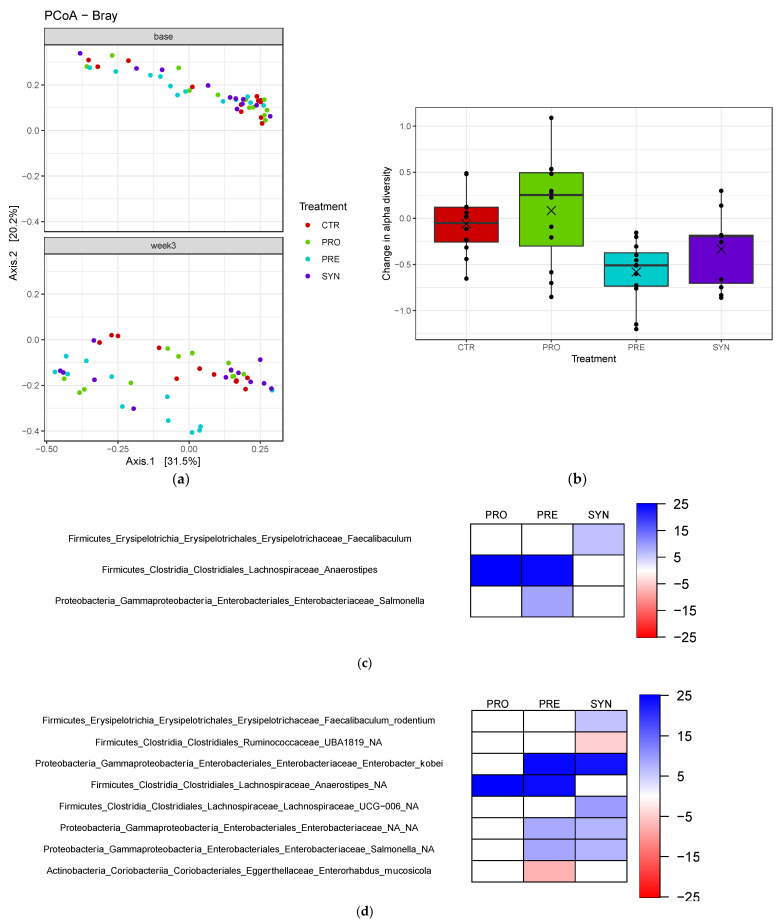
Beta diversity, alpha diversity and gut microbiota composition of BTBR mice at baseline and three weeks post-treatment. (**a**) Beta diversity differences; (**b**) change in alpha diversity. These values are determined by calculating the difference between Shannon diversity at baseline and following the treatment. A negative value represents a decrease in alpha diversity and a positive value represents an increase in diversity. Heatmap highlighting the relative abundances of gut microbiota, at the (**c**) genus and (**d**) species level, of prebiotic-, probiotic- and symbiotic-consuming BTBR mice, which differ significantly from the control group.

Treatment did not significantly affect beta-diversity (between treatment, Figure 6a), but prebiotic consumption significantly reduced alpha-diversity (within treatment) when compared to baseline values (0.5 unit change) (Shannon, *p* = 0.0001) (Figure 6b). Compared to no treatment (CTR), PRE treatment significantly increased the abundance of the genus *Salmonella*, and reduced *Enterorhabdus mucosicola* (*p* < 0.01) (Figure 6c–d). SYN significantly increased *Faecalibaculum rodentium* and *Lachnospiraceae* UCG-006 and reduced *Ruminococcaceae* UBA1819 abundance in fecal matter. The prebiotic or probiotic treatment alone displayed a significant increase in the genus *Anaerostipes*. Prebiotic or synbiotic treatment alone significantly increased the abundances of *Enterobacteriaceae* and an unidentified species of *Salmonella*.

**Figure 7:**
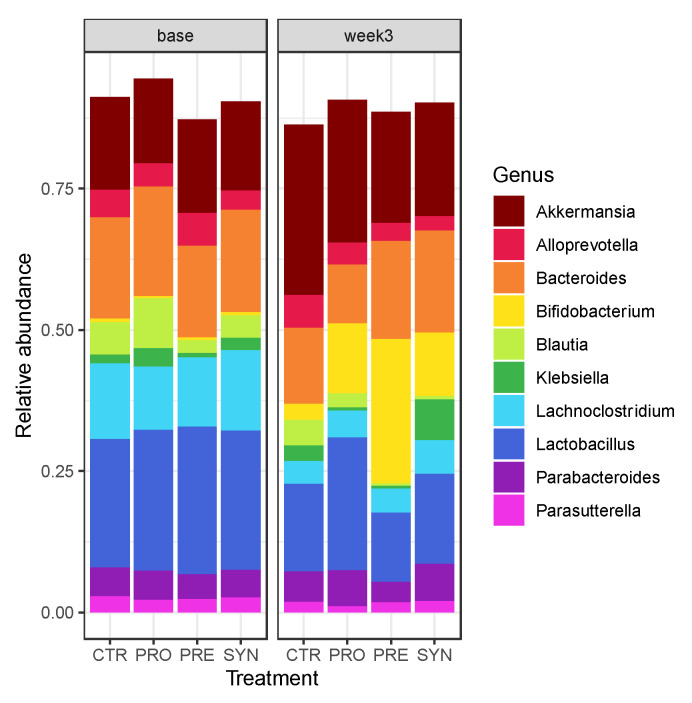
Bar plot of gut microbiota composition at the genus level at baseline and three weeks post-treatment. n = 12/group.

According to the genus-level bar plots, prebiotics increased the abundance of fecal *Bifidobacterium* compared to other groups (Figure 7). Probiotic and synbiotic treatments also increased *Bifidobacterium* levels compared to the control group (Figure 7). Probiotics tended to maintain similar levels of fecal lactobacilli, which was likely driven by the consumption of *L. reuteri*, while other treatment groups showed reduced abundance.

When correlation analysis was performed, the microbiota alpha diversity showed a positive correlational trend with time spent with a stranger mouse (Table 1; r = 0.32, *p* = 0.046), which may be indicative of improved social behavior, and a negative correlational trend with time spent in the empty chamber (Table 1; r = −0.32, *p* = 0.046).

## 4. Discussion

In the current study, we found that probiotic and synbiotic consumption improved social and self-grooming behaviors, as well as improving intestinal permeability. In contrast, we observed that prebiotic consumption tended to worsen sociability measures and increase time spent performing repetitive behaviors, but potentially improved some communication variables. All three treatments altered gut microbiota composition in a manner that may indicate overall improved gut health.

Probiotic and synbiotic consumption significantly improved sociability in BTBR mice, evident from the increased time spent (mean 91.8 s and 70.2 s, respectively) in the chamber containing the novel stranger mouse compared to the empty chamber. Probiotics have shown positive outcomes in terms of social behavior in rodent models of ASD, and limited research reveals similar findings in a clinical population. Sgritta et al. found that *L. reuteri* MM4-1A consumption for four weeks improved sociability in BTBR and *Shank^−/−^* mice [26], consistent with our findings. Similarly, Buffington et al. observed that the offspring of dams consuming a high-fat diet had impaired sociability and drastically reduced abundances of *L. reuteri* [25]. Treating mice for four weeks with *L. reuteri* MM4-1A in drinking water significantly improved sociability [25], suggesting *L. reuteri* has a robust effect on social behavior and is not limited to a single animal model. Improved sociability was also observed in a maternal immune rodent model of ASD when supplementing the diet with 10^10^ CFU *B. fragilis*. Shaaban et al. found sustained improvements in social behavior following the 8-week consumption of a probiotic cocktail [40], suggesting that probiotics may provide a non-invasive therapeutic treatment to improve social behaviors in ASD populations. Overall, our data support the finding that probiotic supplementation may help improve social behaviors in murine models of ASD. However, given the limited findings in human populations, it is important for future clinical research to continue examining the potential role of specific probiotics on social behavior.

Although improvements in sociability were observed with probiotics and synbiotics, consumption of the prebiotic alone (PRE) appears to impair sociability in BTBR mice, indicated by the reduced time (mean 82 s) spent in a chamber containing a stranger mouse compared to in the empty chamber. In contrast to rodent models, Grimaldi et al. found a significant improvement in social behavior and a non-significant reduction in GI symptoms in male and female children with ASD (<12 years of age), following a 6-week supplementation with the prebiotic galactooligosaccharides (1.8 g/day) [41]. Interestingly, greater improvements in sociability were observed when galactooligosaccharides were consumed alongside a restricted diet (casein and/or gluten-free) [41], suggesting that the background diet may be of importance during prebiotic supplementation. This is an interesting observation, given that the sole protein source in our mouse background diet was casein. Furthermore, in a pilot clinical study with n = 8 children diagnosed with autism and GI symptoms, a bovine colostrum product (a source of candidate prebiotic milk oligosaccharides) alone or in combination with *Bifidobacterium longum* supbsp. *infantis* (UCD272) was well-tolerated, and there was some indication of a reduction in aberrant behavior with the colostrum treatment [31]. Thus, further research is warranted in rodent models and human studies to fully elucidate the potential of using prebiotics as a therapeutic treatment in ASD.

Mice treated with prebiotics had increased call durations (mean 41.7 s) and activity (mean 7.5 s), as well as total numbers of USV calls (mean 163.3), which may be indicative of improved communication [33]. Synbiotic mice had greater peak frequencies compared to all groups, and this may reflect an improvement in communication, as wildtype mice have been observed to have greater USV peak frequencies compared to an ASD model (*Shank2^−/−^*) [42]. Although we did not have a wildtype control mouse in our study, given that our goal was to test for differences when compared to our control diet/untreated BTBR mice, Scattoni and colleagues observed that BTBR pups emit USV calls with lower frequencies and shorter durations when separated from their mothers compared to control animals in early life [43]. This is consistent with the phenotype observed in our control BTBR mice. Additionally, Scattoni found that mice had altered USV call-types compared to control groups, resembling atypical communication patterns [43]. When tested as adults, BTBR mice had significantly reduced frequency steps and increased unstructured USV calls compared to control mice [33]. The waveform patterns of USV calls may be sorted and categorized to gain greater insight into their communicative role. Although the specific roles associated with waveform patterns have not been completely elucidated, it has been speculated that they may play an important role in successful mating [44]. Probiotic, prebiotic, and synbiotic mice showed reduced frequencies of short calls (mean 4.7, 2.2 and 0.8, respectively), similar to what was shown for control mice when compared to ASD mice in previous work [42]. Maternal immune activation (MIA) mice also exhibited reduced durations and numbers of USV calls, which are restored upon probiotic treatment (*B. fragilis*) [12]. Although we did not record social behavior during USV calling, Scattoni et al. observed a positive correlation between number of USV calls emitted and social investigation of female mice [33], suggesting greater attraction between mice [45] and perhaps an increased potential for successful reproductive behavior.

We observed reduced bandwidth in mice consuming prebiotic; however, previous research found no difference in USV bandwidth between adult BTBR and B6 control mice [46]. Hanson and Hurley observed that non-ASD male mice alter their USV bandwidth in response in diestrous and estrous female mice, and hypothesized that this may promote reproductive success [47]. Moreover, authors suggest that estrous females may be most attracted to greater-bandwidth USV calls [47]. Future studies may benefit from observing the incidence of specific call-types in connection with social interactions and the reproductive readiness of female mice during USV calling tests, in order to gain greater insight into their roles for both wildtype and BTBR mice.

Probiotic, prebiotic, and synbiotic consumption significantly reduced the number of self-grooming episodes, yet did not impact total time spent grooming. Two-week probiotic treatment with *Lactobacillus rhamnosus* GG also decreased marble-burying behavior (a test of repetitive behavior) in a murine obsessive compulsive model [48]. Additionally, Hsiao et al. found that treating MIA mice with probiotics (*B. fragilis*) reduced the percentage of marbles buried by reducing intestinal permeability and colonic interleukin 6 (IL-6) mRNA expression [12]. Although we did not measure gut cytokines, we did observe an improvement in intestinal permeability alongside reduced repetitive behavior episodes in mice consuming probiotic (PRO and SYN). Future research should consider examining intestinal cytokines to determine whether a possible mechanistic link between intestinal permeability and repetitive behaviors exists.

Intestinal leakiness has been reported in nearly 40% of individuals with ASD [11], and is a likely contributor to the high incidence of gastrointestinal distress in this population [49]. Compromised intestinal permeability allows luminal contents, such as lipopolysaccharides (LPS), to cross the gut epithelium and induce inflammatory responses [50]. Furthermore, intestinal leakiness has been implicated in gastrointestinal conditions, such as irritable bowel syndrome [51], and elevated serum endotoxins observed in severe autism [52] reveal the potential functional outcome of altered intestinal permeability. Probiotic *L. reuteri* consumption has been shown to reduce intestinal permeability [53,54] by increasing the secretion of the intestinotrophic hormone GLP-2 [55], which has independently been shown to improve gut barrier integrity [56]. Improvement in gut leakiness does not seem to be specific to *L. reuteri*, as treatments with *B. fragilis* also improved gut barrier integrity in MIA offspring, a murine environmental autism model [12]. Interestingly, although we saw reductions in intestinal permeability with probiotic consumption, prebiotic consumption did not impact intestinal permeability, despite previous reports of its role in reducing leakiness and stimulating release of GLP-2 [28]. One reason may be the short duration (3 weeks) for which animals were maintained on the diet, which may not have been sufficient to adjust and fully respond to the prebiotic. Although prebiotics have been shown to improve gastrointestinal health, side-effects, such as flatulence and abdominal cramping, have been reported, depending on the dose used [57].

Prebiotic consumption significantly reduced alpha diversity in BTBR mice, which was likely driven by an increase in bifidobacteria, while no change in community structure was observed in prebiotic, synbiotic, or control mice. Altogether, our differential abundance analysis of fecal microbiota revealed an increase in the gut microbiota typically involved in intestinal health, despite little similarity with previous research examining gut microbiota profiles in individuals with ASD and rodent models. For instance, we observed a significant reduction in fecal *Enterorhabdus mucosicola*, which was first discovered in greater abundance in the ileum of a Crohn’s disease mouse model [58]. Moreover, synbiotic consumption significantly increased *Faecalibaculum rodentium*, a lactic acid-producing bacteria [59] that may have anti-obesity potential and may reduce intestinal inflammation [60], while also increasing the abundance of *Lachnospiraceae UCG 006*, a species that has previously been shown to be reduced in a mouse model of irritable bowel syndrome [61]. Butyrate-producer *Anaerostipes* was significantly increased in the probiotic treatment group, and therefore may manifest improvements in gut health, since we saw improved intestinal permeability in BTBR mice consuming a probiotic.

One limitation of the current study is the use of a rodent ASD model to represent clinical autism. Although the BTBR mouse is a well-characterized ASD rodent model of apparent validity, which is well replicated and presents similar neurological changes to those observed in patients with autism, its idiopathy restricts the extrapolation of results to a clinical population. The ASD rodent models allow researchers to determine the mechanistic details of condition pathology and treatment. However, the gold standard for understanding treatment efficacy remains clinical studies. Therefore, future studies should examine the potential effect of probiotic and prebiotic consumption on autism behaviors in various rodent models of ASD, and ultimately in human populations. Additionally, the current study can only observe correlations, thus future studies should perform fecal microbiota transplants to increase the understanding of the microbiota’s role in mediating autism-associated behaviors. Our study did not include a wildtype control mouse, as we were interested in determining if prebiotics and/or probiotics had the potential to improve behaviors, and not necessarily in the “rescuing” of behaviors back to those of a healthy control mouse. We recognize that the lack of a wildtype B6 control mouse limits the interpretability of our results, and we recommend that future studies include this control as a healthy comparator. It would also be of interest to perform a dose–response study with the prebiotic to see if there is a dose that is more beneficial to all outcomes. Finally, future studies would benefit from including female models to determine whether a sex effect exists.

## 5. Conclusions

In summary, *L. reuteri* consumption alone tended to improve sociability, reduce repetitive behavior frequency and grooming bouts, and improve gut barrier integrity. We observed that prebiotic consumption worsened social behavior yet improved communication variables, by increasing the total call activity and number of USV calls and altering the percentage of USV call variables. In the current study, consuming synbiotics improved all three tested behaviors. Finally, we found alterations in the abundances of specific gut microbiota taxa that contribute to improvements in gut health in prebiotic-, probiotic-, and symbiotic-consuming mice, and this may partly explain the improvements observed in ASD-like behavior. Although recent studies have revealed that prebiotic consumption in ASD populations can alter the gut microbiota and improve gastrointestinal functional outcomes, the potential impact of prebiotic consumption on ASD behavior has yet to be properly characterized. Our results suggest that prebiotics may further impair sociability and repetitive behaviors; however, further research in this area is warranted, given that this contrasts with the results of recent human clinical studies that point to improved behaviors and GI symptoms [41]. Additionally, there is a need for future studies to perform fecal microbiota transplants to determine whether changes in gut microbiota composition drive behavioral changes, or whether these are simply consequences of changes in diet.

## Figures and Tables

**Table 1:** Correlation between gut microbiota alpha diversity and behavioral variables.

Variable	Correlation	*p*-Value
Total time in chamber with stranger mouse	0.321	0.046
Total time in empty chamber	−0.321	0.046
Peak spectral density	−0.229	0.117
Frequency bandwidth	−0.094	0.523
USV call duration	−0.198	0.178
Total number of USV calls	−0.066	0.653
Total time self-grooming	0.296	0.068
Self-grooming frequency	0.235	0.150
Repetitive behavior bouts	0.128	0.438

Correlation is considered significant at *p* ≤ 0.01.

## Data Availability

Data are available upon reasonable request from the corresponding author.

## Supplementary Materials

The following is available online at https://www.mdpi.com/article/10.3390/microorganisms9091833/s1, Figure S1: Schematic of the study design.
