# Supplementary figures and images for: Prebiotic, Probiotic, and Synbiotic Consumption Alter Behavioral Variables and Intestinal Permeability and Microbiota in BTBR Mice
Source: Microorganisms. 2021 Aug 29;9(9):1833. doi: 10.3390/microorganisms9091833 (PMC8469248; doi:10.3390/microorganisms9091833)

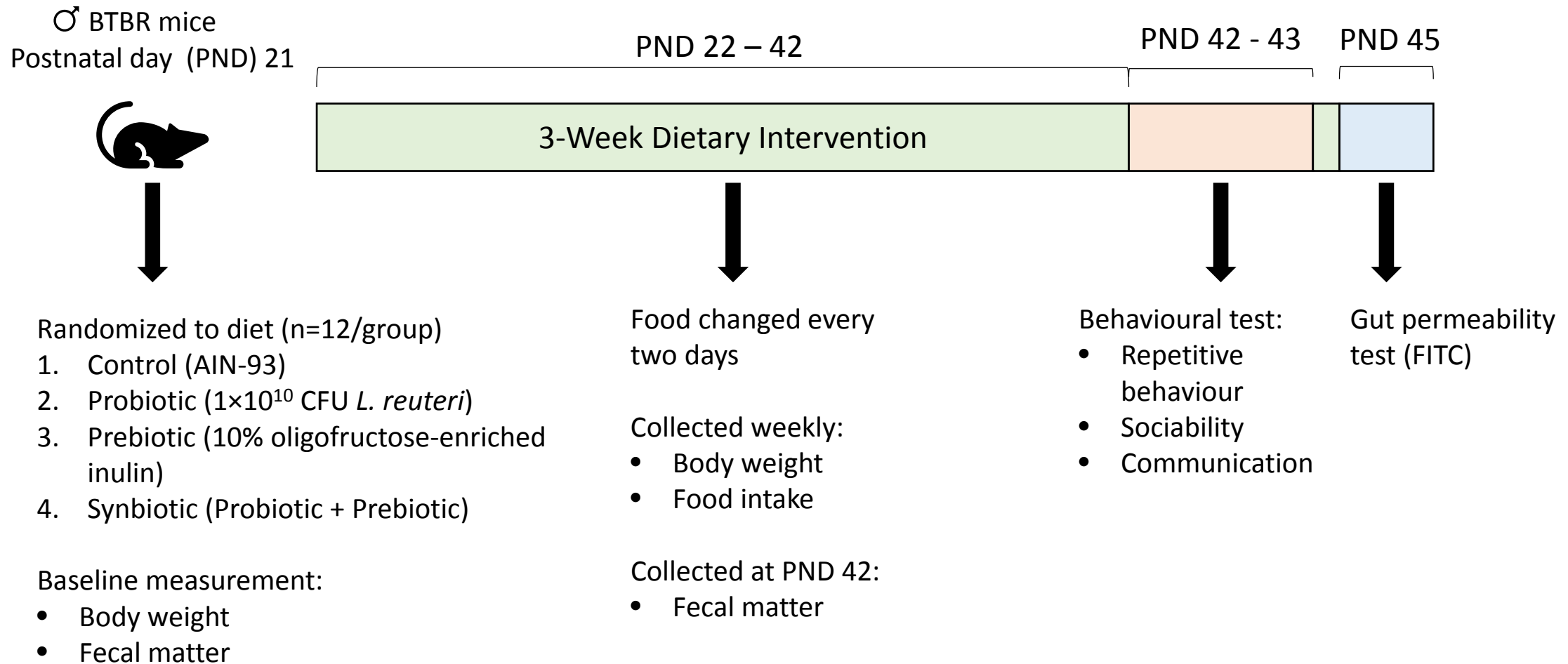

**Figure S1. Schematic of the study design.**

Supplement: Supplementary file 1 [file microorganisms-09-01833-s001.zip › microorganisms-1297405-supplementary.pdf]
